# Role of Dynamical Electron Correlation in the Differences in Bonding between CaAlH_3_ and MgAlH_3_

**DOI:** 10.1021/acs.jpca.1c02422

**Published:** 2021-05-04

**Authors:** Fabio E. Penotti, David L. Cooper, Peter B. Karadakov

**Affiliations:** † Consiglio Nazionale delle Ricerche Istituto di Scienze e Tecnologie Chimiche “Giulio Natta”, Via Golgi 19, I-20133 Milano, MI, Italy; ‡ Department of Chemistry, University of Liverpool, Liverpool L69 7ZD, U.K.; § Department of Chemistry, University of York, Heslington, York YO10 5DD, U.K.

## Abstract

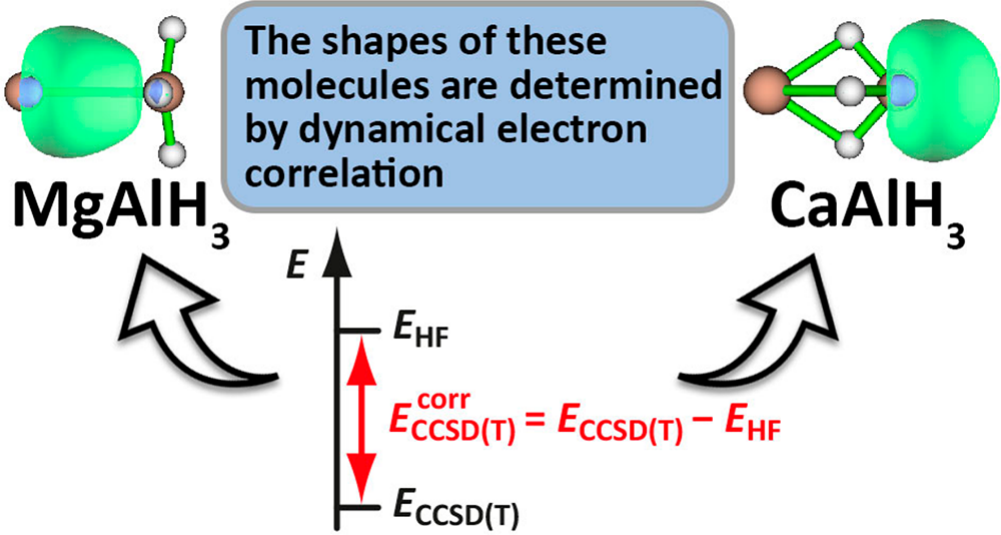

The most important factor behind the intriguing differences between the geometries of the M′AlH_3_ (M′ = Mg, Ca) molecules is shown to be dynamical electron correlation and not intramolecular Coulombic interactions, as previously thought. Spin-coupled generalized valence bond (SCGVB) calculations reveal the different bonding situations in the two molecules at their optimal geometries but do not explain why these geometries differ so much; the solution to this conundrum comes instead from detailed analysis of coupled-cluster (CCSD(T)) energies at model and optimal geometries.

## Introduction

Especially for the ground electronic states of small closed-shell neutral molecules constructed only from main group atoms, for which simple models such as VSEPR (Valence Shell Electron Pair Repulsion) and its various extensions usually work well,^1−5^ it is not very often that dynamical electron correlation is a factor that plays an important role in determining the shape of a neutral molecule and the nature of its bonding in the electronic ground state. We believe that we have identified particularly clear examples of such molecular systems.

**Figure 1:**
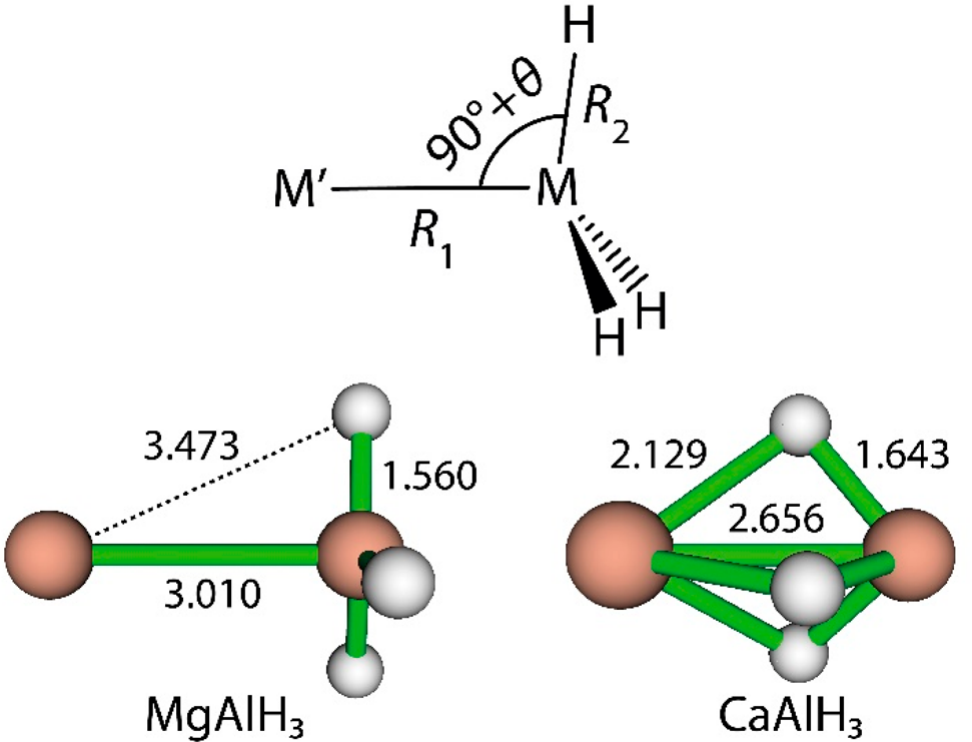
Definition of the geometric parameters *R*_1_, *R*_2_ and θ for *C*_3*v*_ M′MH_3_ species. CCSD(T)/aug-cc-pVQZ geometries of MgAlH_3_ and CaAlH_3_ (see Table 1) with interatomic distances (Å).

Anusiewicz et al.^6^ have recently discovered surprising geometrical features for certain *C*_3*v*_ M′MH_3_ species in which M′ is an alkaline earth atom and M is B, Al, or Ga. As M′ approaches a planar MH_3_ unit along its *C*_3_ axis, a reasonable expectation is of course that some degree of donation would develop of M′(s^2^) valence electrons into the vacant MH_3_(p) orbital. The basic geometric arrangement that would then be anticipated for such a *C*_3*v*_ complex is as shown in Figure 1, in which θ, the deviation of the M′MH bond angle from 90°, is expected to be relatively small and positive, perhaps on the order of +4°, so that the H atoms are directed away from M′. This is indeed what has been found when M′ is Mg but Anusiewicz et al.^6^ observed for the interaction of Ca with AlH_3_ or GaH_3_ that the resulting neutral *C*_3*v*_ complexes have values of θ that are significantly negative, typically on the order of −35°, so that the H atoms are instead directed toward M′; see Figure 1 and Table 1. (Instead of this easily visualized “umbrella angle” θ, we could of course have specified these geometries in terms of the HMHH dihedral angle, in an analogous fashion to interesting previous work on stable anions of M′BH_3_ systems.^7^)

**Table 1:** Geometric Parameters (as Defined in Figure 1) Optimized at the CCSD(T) Level for MgAlH_3_ and CaAlH_3_ with Different Basis Sets^a^

	aug-cc-pVTZ	aug-cc-pVQZ	Literature
MgAlH_3_			
*R*_1_/Å	2.953	3.010	3.003
*R*_2_/Å	1.589	1.560	1.587
ϑ	+3.92°	+3.46°	+3.78°
CaAlH_3_			
*R*_1_/Å	2.749	2.656	2.737
*R*_2_/Å	1.697	1.643	1.706
θ	–34.81°	–36.74°	–35.29°
MgAlH_3_ (Model)			
*R*_1_/Å	2.440	2.380	
*R*_2_/Å	1.693	1.641	
θ (fixed)	–35°	–35°	
CaAlH_3_ (Model)			
*R*_1_/Å	3.280	3.175	
*R*_2_/Å	1.593	1.564	
θ (fixed)	+4°	+4°	

a The values in the “literature” column are taken from Anusiewicz et al.^6^

Much of the specific interest in MgAlH_3_ and CaAlH_3_ has been derived from the fact that such molecules correspond to straightforward functionalizations of alane (AlH_3_), which has numerous applications in organic synthesis as a reducing agent for specific functional groups,^8^ and which is also used as a rocket fuel additive.^9^ Theoretical research on MgAlH_3_ and CaAlH_3_ can be traced back to the late 1980s.^10,11^ For challenges to theory that are presented by these molecules, see ref (12).

The initial idea behind the present work was to use spin-coupled generalized valence bond (SCGVB) calculations for MgAlH_3_ (small positive θ) and CaAlH_3_ (significantly negative θ) to understand *how* the bonding situation differs at these two rather different geometrical arrangements. As part of a first attempt to understand *why* these two systems prefer such different geometries, we also decided to re-examine a suggestion of Anusiewicz et al.^6^ that associated the special geometric characteristics of the Ca systems with an enhanced Coulombic interaction energy between the atomic partial charges. We find, however, that an explanation for the somewhat different geometries is more likely to lie elsewhere.

The structure of this paper is as follows. We outline in the next section the various computational methods that we used. We then present our results and discussion, starting with geometry optimizations and calculations of Coulombic interaction energies between atomic partial charges, before demonstrating how SCGVB theory reveals but does not explain key differences between the two bonding situations, and then identifying an underlying cause for the different optimal geometric arrangements of M′AlH_3_ (M′ = Mg, Ca).

## Computational Methods

Using standard all-electron aug-cc-pVTZ basis sets, the geometries of MgAlH_3_ and CaAlH_3_ were optimized at the CCSD(T) level, correlating all of the electrons. All vibrational frequencies were found to be real, confirming that we had indeed located local minima. The resulting geometric parameters turned out, unsurprisingly, to be similar to those reported by Anusiewicz et al.^6^ To enable fair comparisons of the bonding situations in MgAlH_3_ and CaAlH_3_, we also optimized model geometries for both systems, with θ fixed at −35° for MgAlH_3_ and at +4° for CaAlH_3_. The various CCSD(T) geometry optimizations, which were all carried out using MOLPRO,^13−15^ were subsequently repeated using instead standard aug-cc-pVQZ basis sets, as stored internally by MOLPRO. In particular, the aug-cc-pVQZ basis set for Ca is the one constructed by Hill and Peterson.^16^

Atomic populations were generated at the various CCSD(T)/aug-cc-pVQZ geometries by means of CCSD/aug-cc-pVQZ calculations. From the plethora of rival approaches we chose to use natural population analysis (NPA) partial charges obtained with the natural bond order (NBO) analysis facilities in Gaussian 16^17^ and Voronoi deformation density (VDD) charges,^18^ as implemented in version 3.6 of Multiwfn.^19^ Given that some numerical partial charges (such as those from the standard Mulliken scheme) can be unreliable when using large basis sets with diffuse functions, we decided to use not only aug-cc-pVQZ but also (for the same geometries) the much more modest 6-31G** basis sets that are available at the Basis Set Exchange.^20^ (Note that for 6-31G** we used basis functions in spherical rather than Cartesian form.)

The spin-coupled generalized valence bond (SCGVB) wave functions used here for the eight valence electrons of M′AlH_3_ (M′ = Mg, Ca) take the form
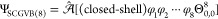
1in which the eight singly occupied nonorthogonal active orbitals, *φ*_*i*_, are fully optimized, without any locality constraints, and the total active space spin function, , is simultaneously optimized as a linear combination of all 14 Kotani spin functions that couple the spins of these eight electrons to yield a state with *S* = 0 and *M*_*S*_ = 0.^21^ Purely for convenience, the doubly occupied inactive closed-shell orbitals for the various SCGVB/aug-cc-pVQZ calculations were taken, without further optimization, from the corresponding CASSCF(8,8)/aug-cc-pVQZ wave functions. We checked that adopting instead RHF or CASSCF(8,11) inactive orbitals leads only to rather trivial changes in the results obtained.

All of the various CASSCF calculations were carried out using the GAMESS-US package^22,23^ and the SCGVB calculations were performed using the generalized multiconfiguration spin-coupled (GMCSC) program developed by Penotti,^24−27^ taking the required integrals over basis functions from GAMESS-US. Pictorial depictions of the resulting SCGVB orbitals were generated using Multiwfn^19^ which was also used for the quantum theory of atoms in molecule (QTAIM) analysis^28^ and, as noted above, for the calculations of VDD partial charges.^18^

## Results and Discussion

The fully optimized *C*_3*v*_ geometries obtained at the CCSD(T) level using aug-cc-pVTZ and aug-cc-pVQZ basis sets (see Table 1) reproduce a key finding of Anusiewicz et al.:^6^ in contrast to the optimal geometry for MgAlH_3_ which has a fairly conventional small positive value for θ of *ca*. +4°, the corresponding angle for CaAlH_3_ is significantly negative (*ca*. −35°). As can be seen from Table 1, switching from aug-cc-pVTZ to the larger aug-cc-pVQZ basis set leads for MgAlH_3_ to small reductions in the values of the AlH distance, *R*_2_, and the angle, θ, with a small increase in the MgAl distance, *R*_1_. It turns out that our two sets of geometric parameters for MgAlH_3_ straddle those reported previously.^6^ The corresponding improvement in the basis set for CaAlH_3_ leads to small reductions in *R*_1_ and *R*_2_, with a parallel increase in θ. Although here again the two sets of *R*_1_ and θ values straddle those reported previously,^6^ which were based on calculations that used a pseudopotential for Ca, our values of *R*_2_ turn out to be slightly smaller.

Whereas the optimal *C*_3*v*_ geometry of MgAlH_3_ is characterized by a relatively large MgAl separation, that for CaAlH_3_ features a relatively short CaAl separation as well as AlH distances that are slightly longer than in MgAlH_3_. As a first step toward trying to understand these differences, we constructed a model geometry for MgAlH_3_ in which θ was fixed at −35° (comparable to that in the optimal structure for CaAlH_3_). According to CCSD(T)/aug-cc-pVTZ calculations, the energetic cost relative to the fully optimized geometry of fixing θ in this way, without reoptimization of *R*_1_ and *R*_2_, is 23.6 kcal/mol. Keeping these fixed values of *R*_2_ and θ, we then reoptimized *R*_1_, finding a somewhat smaller value of 2.435 Å, accompanied by an energy lowering of 15.5 kcal/mol. Finally, we reoptimized both *R*_1_ and *R*_2_ with θ still fixed at −35°. This resulted in only a very small additional change in *R*_1_, but in a significant increase in *R*_2_ to 1.693 Å and an energy lowering of 3.6 kcal/mol. It is striking that the resulting geometry (see Table 1) features AlH distances that are now much the same as those in CaAlH_3_ and that the MgAl separation is also now somewhat smaller than that for the optimal geometry. Overall, we find at this level of theory that the fully optimized geometry for MgAlH_3_ is preferred relative to the optimized model structure with fixed θ = −35° by 4.5 kcal/mol. (Note that we have simply compared the CCSD(T) energies, without attempting any corrections for differences in the zero-point vibrational energies for different θ values. The various CCSD(T) energies for all these geometries are reported in Table S1 in the Supporting Information.)

It did of course seem rather worthwhile to repeat this exercise for CaAlH_3_, this time changing θ to a fixed value of +4° (comparable to that in the optimal structure for MgAlH_3_). It was our expectation that the AlH distances (*R*_2_) would end up being similar to those observed for the optimal MgAlH_3_ structure and also that the CaAl distance (*R*_1_) would become somewhat longer than in the optimal CaAlH_3_ structure. According to the CCSD(T)/aug-cc-pVTZ calculations, the energetic cost for CaAlH_3_ of fixing θ = +4° without reoptimization of *R*_1_ and *R*_2_ is 21.3 kcal/mol. Subsequent optimization of *R*_1_ with fixed θ and *R*_2_ leads to an energy lowering of 6.6 kcal/mol, with *R*_1_ = 3.244 Å. Finally, fixing only θ = +4°, we find *R*_1_ = 3.280 Å, *R*_2_ = 1.593 Å, and a further energy improvement of 4.2 kcal/mol. All in all, our expectations have been realized (see Table 1): the AlH distances for the model geometry of CaAlH_3_ with fixed θ = +4° are similar to those in the optimal MgAlH_3_ structure, and similarly, the CaAl separation is somewhat larger than in the optimal geometry of CaAlH_3_. Overall, at this level of theory, the optimal geometry for CaAlH_3_ turns out to be preferred relative to the optimized model structure with fixed θ = +4° by 10.5 kcal/mol (again simply comparing the CCSD(T) energies).

Also shown in Table 1 are the corresponding model geometries for MgAlH_3_ and CaAlH_3_ that were generated using instead CCSD(T)/aug-cc-pVQZ calculations. (The various energies are reported in Table S1 in the Supporting Information.) The overall patterns in the values of *R*_1_ and *R*_2_ are clearly the same as we observed with the smaller basis set. For the M′AlH_3_ geometries with θ ∼ +4°, switching M′ from Mg to Ca has little effect on the AlH distances, but it increases the M′Al separation by 0.16–0.33 Å, depending on the quality of the basis set. Comparing instead the two geometries with θ ∼ −35°, it is clear that switching M′ from Mg to Ca again has little effect on the (slightly longer) AlH distances but it increases the M′Al separation by *ca*. 0.3 Å.

We could of course imagine that the process of forming M′AlH_3_ involves an initial symmetrical distortion of AlH_3_, so as to adopt the same geometry as in the M′AlH_3_ complex, and then the further changes due to the interaction of this deformed AlH_3_ unit with the M′ atom. It is clear from the CCSD(T)/aug-cc-pVQZ energies of various geometries of symmetrically distorted AlH_3_ (as reported in Table S2 in the Supporting Information) that most of the deformation energy is associated with the change in θ rather than with the increased AlH separation. Comparing the CCSD(T)/aug-cc-pVQZ energies for M′AlH_3_ (as listed in Table S1 in the Supporting Information, without any corrections for vibrational energies), we find that our model geometry for MgAlH_3_ with θ = −35° is disfavored relative to the optimal one with θ ∼ +4° by 4.6 kcal/mol. In this case a cost of 58.8 kcal/mol for further distortion of the AlH_3_ moiety is only partially compensated by an increase in the interaction energy with the Mg atom of 54.1 kcal/mol. The situation is of course entirely different in CaAlH_3_, for which the optimal geometry with θ ∼ −35° is favored relative to the model geometry (θ = +4°) by 21.1 kcal/mol at this level of theory. It turns out that this overall energy difference corresponds to a cost of 64.7 kcal/mol for the further distortion of the AlH_3_ moiety being more than compensated by an increase of 85.8 kcal/mol for the interaction energy with the Ca atom. Relative to the situation for θ ∼ +4°, the enhanced interaction for θ ∼ −35° between the deformed AlH_3_ moiety and the M′ atom clearly increases by more than 50% (54.1–85.8 kcal/mol) upon switching from Mg to Ca.

Investigating the energetic differences between MgAlH_3_ and CaAlH_3_, Anusiewicz et al.^6^ used sets of point charges located at nuclear positions in order to assess the relative values of the Coulombic attraction energies between positively and negatively charged centers. Using their M′H separations and the NPA partial charges obtained from NBO analysis, they showed that the Coulombic interaction energy between the net positive charge on the M′ atom and the net negative charges on each of the three H atoms is substantially more favorable for CaAlH_3_ with θ ∼ −35° than it is for MgAlH_3_ with θ ∼ +4°. They identified this difference as being key for rationalizing the preference shown by CaAlH_3_ for the geometry with significantly negative θ. Certainly this special geometric characteristic does enhance the Coulombic attraction between the net positive charge on the Ca atom and the net negative charges on the H atoms. All of this is of course entirely plausible but, nonetheless, it is informative also to compare two M′AlH_3_ species that have much the same value of θ, whether it is ∼+4° or ∼−35°. With this in mind, we decided for our various CCSD(T)/aug-cc-pVQZ geometries to use atomic populations (CCSD level), together with the calculated M′Al and M′H distances, so as to assess the relative values of Coulombic interaction energies, in a fashion very similar to that of Anusiewicz et al.^6^

Comparing the atomic populations (CCSD level) calculated using aug-cc-pVQZ and 6-31G** basis sets, we observe that the NPA charges show only modest basis set dependence and the same is true for the VDD charges but that the corresponding sets of VDD and NPA charges are nonetheless rather different from one another (see Table S3 in the Supporting Information). The NPA charge on Al is typically on the order of about double the corresponding VDD charge and, at the θ ∼ −35° geometries, the NPA charge on M′ is on the order of about 3 times the corresponding VDD charge. The Mulliken charges (6-31G** basis set) resemble most those from the VDD scheme. We then examined the simple point-charge Coulombic energies both for the interaction of M′ with just the three H atoms, as was done by Anusiewicz et al.,^6^ and for the interaction of M′ with all of the AlH_3_ moiety.

Clearly the significant differences between the NPA and VDD (or Mulliken) estimates of the degree of charge separation must have a big impact on the calculated Coulombic energies. Nonetheless, we find consistently, whether we consider the M′···H_3_ or M′···AlH_3_ interactions, that these Coulombic interactions are indeed more attractive for CaAlH_3_ with θ ∼ −35° than they are for MgAlH_3_ with θ ∼ +4° (see Tables S4 and S5 in the Supporting Information). Unsurprisingly, the magnitude of this preference varies significantly with the particular choice of partial charges. However, we also observe for the two M′AlH_3_ geometries with θ ∼ +4° that the Coulombic interaction energies are mostly rather similar to one another for any given mode of calculation. It turns out that such an observation also holds to a fair degree for the M′AlH_3_ geometries with θ ∼ −35°. On the whole, the change in θ appears to be far more important for the Coulombic interaction energy between atomic partial charges than is changing the M′ atom. As a consequence, even without repeating this simple analysis with many different sets of partial charges, it does unfortunately already seem slightly questionable to invoke differences in M′···H_3_ Coulombic attractions when rationalizing the preference of the Mg system for small positive θ and of its Ca counterpart for significantly negative θ.

**Figure 2:**
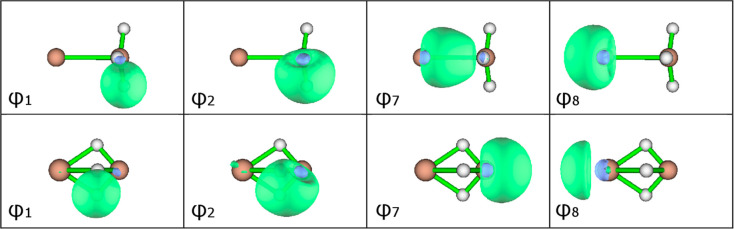
Symmetry-unique SCGVB orbitals for MgAlH_3_ (top row) and CaAlH_3_ (bottom row) at the optimal geometries of these two molecules.

In order to reveal how the bonding situations differ between MgAlH_3_ and CaAlH_3_ at their optimal CCSD(T)/aug-cc-pVQZ geometries, we then turned to the results of SCGVB/aug-cc-pVQZ calculations. The description that emerges for MgAlH_3_ at its optimal geometry features three symmetry-equivalent pairs, one for each AlH bond, with, in each case, one orbital (see φ_1_ in top row of Figure 2) that is semilocalized on H, whereas the corresponding Al-based orbital (see φ_2_ in top row of Figure 2) shows rather significant deformation toward H, consistent with the electronegativity differences. The two orbitals in each pair have an overlap of 0.81 (see Table 2), and the corresponding electron spins are overwhelmingly singlet coupled. Taken together, these observations indicate the presence of three somewhat unsurprising AlH bonds, very much as we might have expected for this geometrical arrangement. There are much smaller overlaps (see Table 2) between SCGVB orbitals that are associated with different AlH bonds. To a first approximation, we observe from the forms of orbitals φ_7_ and φ_8_ that the doubly occupied Mg(3s^2^) orbital splits into separate s_–_ and s_+_ lobe orbitals that are concentrated in opposite directions along the *C*_3_ axis. Unlike the s_–_ orbital, which is concentrated away from Al (see φ_8_ in top row of Figure 2) and mostly retains its basic form, the corresponding s_+_ function deforms/distorts significantly toward the Al center (see φ_7_ in top row of Figure 2), thereby also transferring charge to the AlH_3_ moiety. Orbitals φ_7_ and φ_8_ do, though, retain an overlap of 0.59. The overall active space spin function is dominated by the perfect pairing mode of spin coupling, with a weight of a little more than 99.7%.

**Table 2:** SCGVB Orbital Overlaps for MgAlH_3_ at Its Optimal CCSD(T)/aug-cc-pVQZ Geometry

	φ_1_	φ_2_	φ_3_	φ_4_	φ_5_	φ_6_	φ_7_	φ_8_
φ_1_	1							
φ_2_	0.81	1						
φ_3_	0.06	0.13	1					
φ_4_	0.13	0.30	0.81	1				
φ_5_	0.06	0.13	0.06	0.13	1			
φ_6_	0.13	0.30	0.13	0.30	0.81	1		
φ_7_	0.10	0.22	0.10	0.22	0.10	0.22	1	
φ_8_	0.02	0.02	0.02	0.02	0.02	0.02	0.59	1

The SCGVB description of CaAlH_3_ at its optimal geometry is dramatically different although there are again three symmetry-equivalent pairs, this time with one pair in each CaHAl bridge. One of the orbitals in each pair (see φ_1_ in bottom row of Figure 2) remains semilocalized on the H atom. Its partner (see φ_2_ in bottom row of Figure 2) also has significant amplitude on the H atom, suggestive of H^–^ character, but this orbital extends all of the way from the Al center across the H atom toward the Ca atom. The two orbitals in each pair have an overlap of 0.82 (see Table 3) so that, taken together with the form of orbital φ_2_ and a weight of a little more than 99.6% for the perfect pairing mode of spin coupling, this appears to suggest a degree of three-center, two-electron interactions across the polar CaHAl bridges. We observe from the depictions in the bottom row of Figure 2 that orbital φ_8_ does still have the basic appearance of an s_–_ lobe orbital but that φ_7_ takes a somewhat different form from that in MgAlH_3_. Instead of an s_+_ lobe function that is heavily deformed toward the Al atom, it has effectively transferred across to the Al atom, taking the form of an Al lobe orbital that is concentrated in the direction that points away from, rather than into, the bridging region. All of this is of course consistent with higher magnitudes for the atomic partial charges in this molecule. The magnitude of the overlap between φ_7_ and φ_8_ (see Table 3) is relatively small, being 0.21.

**Table 3:** SCGVB Orbital Overlaps for CaAlH_3_ at Its Optimal CCSD(T)/aug-cc-pVQZ Geometry

	φ_1_	φ_2_	φ_3_	φ_4_	φ_5_	φ_6_	φ_7_	φ_8_
φ_1_	1							
φ_2_	0.82	1						
φ_3_	0.09	0.15	1					
φ_4_	0.15	0.27	0.82	1				
φ_5_	0.09	0.15	0.09	0.15	1			
φ_6_	0.15	0.27	0.15	0.27	0.82	1		
φ_7_	0.12	0.32	0.12	0.32	0.12	0.32	1	
φ_8_	0.06	0.05	0.06	0.05	0.06	0.05	–0.21	1

It proves useful at this stage also to examine the results of QTAIM analyses of these SCGVB wave functions. Reasonably linear bond paths from Al to H are observed for MgAlH_3_, and moreover, there is an entirely straightforward bond path from Mg to Al, passing through a somewhat ordinary bond critical point. On the other hand, the corresponding analysis for CaAlH_3_ reveals instead distinctly curved bond paths between the atoms in each CaAlH bridge. Furthermore, instead of a bond critical point and a corresponding bond path, the QTAIM analysis detects the presence of a cage critical point between the Ca and Al atoms. (That cage critical point is in effect surrounded by three ring critical points in a plane that is perpendicular to the *C*_3_ axis, with each of the cage critical point to ring critical point directions bisecting a pair of CaAlH bridges.) All in all, visual inspection of the SCGVB solutions as well as the outcome of the QTAIM analysis appears to suggest the absence of any significant degree of direct Ca to Al bonding interactions in this molecule.

The SCGVB calculations, as well as the QTAIM analyses, clearly reveal in a very straightforward and highly visual manner *how* the bonding situation for CaAlH_3_ at its optimal geometry differs from that for MgAlH_3_ at its optimal geometry. However, a deeper conundrum remains unanswered: *why* do these two molecules have such different optimal geometries? Seeking a plausible answer, it might seem tempting at this stage to carry out more intimate inspections of the two SCGVB solutions, looking for appropriate differences. However, as we demonstrated above for differences of Coulombic interaction energies between partial charges, it can prove important to attempt to consider separately the change in the angle θ from the replacement of Mg by Ca. Use of the model geometries with optimized values of *R*_1_ and *R*_2_, but with θ fixed at −35° for MgAlH_3_ and at +4° for CaAlH_3_, showed in that case that the change from Mg to Ca for a given θ was far less important than the change in geometry. Accordingly, we also carried out SCGVB calculations for MgAlH_3_ and CaAlH_3_ at our CCSD(T)/aug-cc-pVQZ model geometries. We find that each of the SCGVB solutions in the present work accounts for almost 99% of the electron correlation that is provided by the corresponding CASSCF(8,8) wave function (Table S6 in the Supporting Information). To a first approximation, the resulting SCGVB active orbitals for MgAlH_3_ with θ = −35° (top row of Figure S1 in the Supporting Information) and the overlaps between them (Table S7 in the Supporting Information) are somewhat reminiscent of those for CaAlH_3_ at its optimal geometry. Similarly, the corresponding results for CaAlH_3_ with θ = +4° (Table S8 and bottom row of Figure S1 in the Supporting Information) have a fair amount in common with those for MgAlH_3_ at its optimal geometry. There are of course some differences that can be linked to the change from Mg(3s^2^) to Ca(4s^2^) and there is also some evidence for the incorporation of a small degree of Ca(3d_*z*^2^_) character, as might have been anticipated from the study of Fernández et al.,^29^ but it does not seem likely that these features could be sufficient on their own to explain the very different geometric preferences of the MgAlH_3_ and CaAlH_3_ molecules. Unsurprisingly, we also find that QTAIM analyses of the SCGVB wave functions for each of the model geometries reveals the same basic pattern of critical points and bond paths as for the optimal geometry with the comparable value of θ. (Note that we also found for all four of the CCSD(T)/aug-cc-pVQZ geometries that switching from SCGVB to RHF or CCSD densities made essentially no difference to the resulting patterns of critical points and bond paths.)

It does in fact now turn out that an underlying cause for the rather different optimal geometric arrangements of M′AlH_3_ (M′ = Mg, Ca) might actually have been hidden in plain sight all along. To show that this is the case, it proves instructive to examine the simple energy differences (Δ*E*) for a given molecule between the model and optimal CCSD(T)/aug-cc-pVQZ geometries, as calculated at various levels of theory using the aug-cc-pVQZ basis set. A selection of such results is reported in Table 4, in which negative values of Δ*E* for a given molecule indicate that the geometry with θ ∼ −35° is preferred whereas positive values indicate that it is the geometry with θ ∼ +4° that gives the lower energy. (Additional values are reported in Table S9 in the Supporting Information.)

**Table 4:** Simple Energy Differences (Δ*E*) between the θ ∼ −35° and θ ∼ +4° CCSD(T)/aug cc pVQZ Geometries, as Calculated for a Given Molecule at Various Levels of Theory Using the aug cc pVQZ Basis Set^a^

	Δ*E* (kcal/mol)	
method	MgAlH_3_	CaAlH_3_
RHF	31.2	12.2
SCGVB	15.4	0.7
CASSCF(8,8)	15.1	0.6
B3LYP	12.9	–6.8
CCSD	9.0	–16.6
CCSD(T)	4.6	–21.1

a Negative values of Δ*E* indicate a preference for the θ ∼ −35° geometry.

The various energy differences Δ*E* between the two geometries for a given molecule seem to send a very clear message. Although the optimal geometry for MgAlH_3_ is preferred at the RHF level by 31.2 kcal/mol, this energy difference is approximately halved when account is taken of nondynamical electron correlation, whether by means of SCGVB or CASSCF calculations. The incorporation of dynamical electron correlation leads to even smaller values, so that at the CCSD(T) level the energy difference is just 4.6 kcal/mol, i.e., less than 15% of the RHF value.

The consequences of taking account of electron correlation are even more dramatic in the case of CaAlH_3_, for which the model geometry with θ ∼ +4° is the preferred one at the RHF level by 12.2 kcal/mol. Taking account of nondynamical electron correlation, whether by means of SCGVB or CASSCF calculations, reduces this preference by *ca*. 11.5 kcal/mol so that although the model geometry is still the preferred one, the energy difference is now less than 1 kcal/mol. Inclusion of dynamical electron correlation tips the balance further away from the model geometry, so that it is indeed now the optimal geometry which becomes the preferred one, with the value of Δ*E* reaching −21.1 kcal/mol at the CCSD(T) level. Ultimately, it seems for a given molecule that electron correlation, especially dynamical correlation, lowers the energy of the geometries with θ ∼ −35° somewhat more than it does those with θ ∼ +4°, probably on account of less spatial separation between electron pairs. For MgAlH_3_ this leads to a reduction in the preference for the optimal θ ∼ +4° geometry by 26.6 kcal/mol, from 31.2 kcal/mol at the RHF level to 4.6 kcal/mol for CCSD(T). A reduction of this magnitude for CaAlH_3_, for which Δ*E* is 12.2 kcal/mol at the RHF level, would already have been enough to switch the energetic ordering of the two geometries. Indeed, the reduction in Δ*E* for CaAlH_3_ from RHF to CCSD(T) is larger than that for MgAlH_3_, being instead 33.3 kcal/mol. Such an extra 6.8 kcal/mol would in fact have been enough in the case of MgAlH_3_ to make the model geometry with θ = −35° the preferred one.

Whereas the Coulombic interaction energies consistently favor the geometries with θ ∼ −35° over those with θ ∼ +4°, it is the latter that are the preferred ones at the RHF level. As we have seen, the incorporation of electron correlation brings down the energies of the θ ∼ −35° geometries relative to those of the θ ∼ +4° geometries such that the lowering at the CCSD(T) level is sufficient for CaAlH_3_, but not quite enough in the case of MgAlH_3_ to switch the energetic ordering of the two geometries. We might though still ponder the magnitude of the differences in the RHF Δ*E* values: 31.2 kcal/mol for MgAlH_3_ is reduced to 12.2 kcal/mol for CaAlH_3_, a difference of 19.0 kcal/mol. The corresponding changes in our simple estimates of the Coulombic interaction energies between RHF VDD atomic partial charges turn out to be 6.9 kcal/mol for M′···H_3_ and 15.9 kcal/mol for M′···AlH_3_. These values are at least of the right magnitude.

Certainly, it remains true that enhanced Coulombic interaction energies between atomic partial charges could play a role in explanations of the different geometric preferences of these M′AlH_3_ (M′ = Mg, Ca) molecules and the same might be said for the rather different bonding arrangements that are revealed by SCGVB theory. Nonetheless, the most important factor ultimately turns out to be electron correlation. Although this is entirely straightforward, it is also mildly disappointing because it seems to have denied us a simple highly visual explanation that is based on traditional chemical concepts such as partial charges and differences in the bonding situations.

The strong dominance of the perfect pairing mode of spin coupling in the overall active space spin functions from the SCGVB descriptions of both MgAlH_3_ and CaAlH_3_ indicates that these are essentially closed-shell molecules for which it is completely appropriate to use standard coupled-cluster methods based on a closed-shell Hartree–Fock reference. While use of more elaborate coupled-cluster methods and/or even larger basis sets could lead to changes in the energy differences reported in Table 4, the quality of the CCSD(T)/aug-cc-pVQZ combination is sufficient to guarantee that any such changes would be relatively minor and would not affect our explanation of the differences in bonding between MgAlH_3_ and CaAlH_3_.

## Conclusions

We were certainly attracted to the suggestions of a link between Coulombic interaction energies and the somewhat different geometric characteristics of the M′AlH_3_ (M′ = Mg, Ca) molecules.^6^ However, our confidence in such an explanation was dented when we examined also a model geometry for CaAlH_3_ with an angle, θ = +4°, comparable to that in the optimal geometry of MgAlH_3_ and, similarly, a model geometry for MgAlH_3_ with an angle, θ = −35°, comparable to that in the optimal geometry of CaAlH_3_. Whether we consider changes to the M′···H_3_ or M′···AlH_3_ Coulombic interaction energies between atomic partial charges, we find the change in θ to be far more important than is the replacement of Mg by Ca.

The SCGVB calculations reported here reveal a rather straightforward description for the optimal geometry of MgAlH_3_. We may envisage the interaction of the Mg atom with the AlH_3_ moiety in terms of the doubly occupied Mg(3s^2^) orbital splitting into separate s_–_ and s_+_ lobe orbitals that are directed away from and toward, respectively, the Al atom. Unlike the corresponding s_–_ orbital, which mostly retains its basic form, the s_+_ function deforms/distorts significantly toward the Al center, thereby also transferring charge to the AlH_3_ moiety. The forms of the various SCGVB active orbitals and the overlaps between them, the dominance of the perfect pairing mode of spin coupling, and the observed pattern of QTAIM critical points and bond paths all point to the presence of direct MgAl and AlH bonding.

Turning now to the SCGVB description for the optimal geometry of CaAlH_3_, we observe instead of a heavily deformed s_+_ lobe function on Ca that there is a lobe function on the Al center directed away from, rather than into, the bonding region. In this case, the forms of the various SCGVB active orbitals and the overlaps between them, the dominance of the perfect pairing mode of spin coupling, and the observed pattern of QTAIM critical points and bond paths indicate that the bonding is focused in the polar CaHAl bridges with a degree of three-center, two-electron interactions but no significant degree of direct CaAl bonding.

The various SCGVB calculations certainly show *how* the bonding situation in CaAlH_3_ differs from that in MgAlH_3_ when they are both at their optimal geometries but not *why* the geometries differ. That this is so becomes apparent when examining also the SCGVB descriptions of the bonding for the model geometries of MgAlH_3_ and CaAlH_3_. Reminiscent of our findings for the Coulombic interaction energies between atomic partial charges, we observe that changing the geometry from θ ∼ +4° to θ ∼ −35° plays a much larger role in determining the nature of the SCGVB solutions than does the replacement of Mg by Ca.

Looking instead at energy differences between model and optimal CCSD(T)/aug-cc-pVQZ geometries, we find that the more standard geometric arrangement with θ ∼ +4° is in fact the preferred one at the RHF/aug-cc-pVQZ level for both of these molecules. It turns out that the incorporation of electron correlation, especially dynamical correlation, brings down the energies of the geometries with θ ∼ −35° relative to those with θ ∼ +4°, on account of reduced spatial separation between electron pairs. In the end, the lowering at the CCSD(T) level turns out to be sufficient for CaAlH_3_, but not quite enough in the case of MgAlH_3_, to switch the energetic ordering, with the consequence being that these two molecules exhibit dramatically different optimal geometries.

## Supporting Information Available

CCSD(T) energies for various geometries of M′AlH_3_ and AlH_3_; CCSD atomic populations for M′AlH_3_; Coulombic interaction energies; RHF, SCGVB, and CASSCF energies for M′AlH_3_; SCGVB orbitals and orbital overlaps for M′AlH_3_ at model geometries; symmetry-unique SCGVB orbitals; simple energy differences between θ ∼ −35° and θ ∼ +4° geometries (PDF)
