# Supplementary material for: Role of Dynamical Electron Correlation in the Differences in Bonding between CaAlH3 and MgAlH3
Source: J Phys Chem A. 2021 May 4;125(18):3912–9. doi: 10.1021/acs.jpca.1c02422 (PMC9297285; doi:10.1021/acs.jpca.1c02422)
Supplement: Supplementary file 1 — jp1c02422_si_001.pdf [file jp1c02422_si_001.pdf]

Supporting Information

Role of Dynamical Electron Correlation in the Differences in Bonding Between $CaAlH_3$ and $MgAlH_3$

Fabio E. Penotti†, David L. Cooper‡ and Peter B. Karadakov§

†*Consiglio Nazionale delle Ricerche, Istituto di Scienze e Tecnologie Chimiche "Giulio Natta", Via Golgi 19, I–20133 Milano MI, Italy*

‡*Department of Chemistry, University of Liverpool, Liverpool L69 7ZD, UK*

§*Department of Chemistry, University of York, Heslington, York YO10 5DD, UK*

Corresponding authors: Fabio E. Penotti – Email: f.penotti@gmail.com; David L. Cooper – Email: dlc@liverpool.ac.uk

Table of Contents

Table S1	CCSD(T) energies for various geometries of M′AlH_3	S3
Table S2	CCSD(T) energies for symmetrically distorted AlH_3	S3
Table S3	CCSD atomic populations for M′AlH_3	S4
Table S4	M′…H_3 Coulombic interaction energies for M′AlH_3	S4
Table S5	M′…AlH_3 Coulombic interaction energies for M′AlH_3	S5
Table S6	RHF, SCGVB and CASSCF energies for M′AlH_3	S5
Table S7	SCGVB orbital overlaps for $MgAlH_3$ at its model θ = −35° geometry	S6
Table S8	SCGVB orbital overlaps for $CaAlH_3$ at its model θ = +4° geometry	S6
Figure S1	Symmetry-unique SCGVB orbitals at model geometries	S6
Table S9	Simple energy differences between θ ~ −35° and θ ~ +4° geometries	S7

Table S1 CCSD(T) energies (in hartree) for various C_{3v} geometries of $MgAlH_3$ and $CaAlH_3$.

	R_1/Å	R_2/Å	θ	Energy	$R_{M'H}$/Å
**$MgAlH_3$**					
aug-cc-pVTZ	2.952711	1.589493	+3.92063°	−443.5185795	3.447724
	2.952711	1.589493	−35°	−443.4809235	2.420959
	2.434514	1.589493	−35°	−443.5056371	2.003565
	2.439984	1.692880	−35°	−443.5114212	2.020133
aug-cc-pVQZ	3.010287	1.560233	+3.46140°	−443.5302167	3.473226
	2.379944	1.640973	−35°	−443.5228466	1.968961
**$CaAlH_3$**					
aug-cc-pVTZ	2.749063	1.696520	−34.80580°	−920.8255329	2.260823
	2.749063	1.696520	+4°	−920.7915749	3.329594
	3.243867	1.696520	+4°	−920.8021233	3.764125
	3.279750	1.592916	+4°	−920.8087955	3.744731
aug-cc-pVQZ	2.655714	1.642523	−36.73782°	−920.8791522	2.128923
	3.174663	1.563659	+4°	−920.8455061	3.635391

Table S2 CCSD(T)/aug-cc-pVQZ energies (in hartree) and relative energies ΔE_d (in kcal/mol) for symmetrically distorted AlH_3.

θ	R_{AlH}/Å	Energy	ΔE_d
0°	1.55301308	−243.8378853	0
3.46139925°	1.56023279	−243.8369119	0.61
4°	1.56365923	−243.8365491	0.84
35°	1.64097258	−243.7432534	59.38
36.73781912°	1.64252250	−243.7334681	65.52
0°	1.56023279	−243.8378360	0.03
0°	1.56365923	−243.8377780	0.07
0°	1.64097258	−243.8312644	4.15
0°	1.64252250	−243.8310430	4.29

Table S3 CCSD atomic populations at the CCSD(T)/aug-cc-pVQZ C_{3v} geometries of M′AlH_3 (see Table S1), except for values taken from Ref. 1.

Populations	θ ~ +4°				θ ~ −35°			
	$MgAlH_3$		$CaAlH_3$		$MgAlH_3$		$CaAlH_3$	
	Mg	Al	Ca	Al	Mg	Al	Ca	Al
Ref. 1	0.300	0.854					0.975	0.453
aug-cc-pVQZ								
NPA	0.293	0.883	0.462	0.693	1.002	0.444	1.178	0.385
VDD	0.206	0.379	0.272	0.323	0.341	0.212	0.397	0.139
6-31G**								
NPA	0.281	0.832	0.401	0.680	0.945	0.427	1.034	0.467
VDD	0.191	0.377	0.247	0.330	0.313	0.217	0.335	0.179
Mulliken	0.103	0.437	0.146	0.391	0.180	0.331	0.363	0.365

Table S4 M′…H_3 Coulombic interaction energies (in eV) at the C_{3v} CCSD(T)/aug-cc-pVQZ geometries of M′AlH_3 (see Table S1) calculated using CCSD atomic populations (see Table S3), except for the values taken from Ref. 1.

Populations	θ ~ +4°		θ ~ −35°	
	$MgAlH_3$	$CaAlH_3$	$MgAlH_3$	$CaAlH_3$
Ref. 1	−1.43			−8.96
aug-cc-pVQZ				
NPA	−1.43	−2.11	−10.59	−12.45
VDD	−0.50	−0.64	−1.38	−1.44
6-31G**				
NPA	−1.29	−1.72	−9.48	−10.49
VDD	−0.45	−0.56	−1.22	−1.16
Mulliken	−0.23	−0.31	−0.67	−1.79

Table S5 M′…AlH_3 Coulombic interaction energies (in eV) at the C_{3v} CCSD(T)/aug-cc-pVQZ geometries of M′AlH_3 (see Table S1) calculated using CCSD atomic populations (Table S3), except for the values based on data in Ref. 1.

Populations	θ ~ +4°		θ ~ −35°	
	$MgAlH_3$	$CaAlH_3$	$MgAlH_3$	$CaAlH_3$
Ref. 1	−0.20			−6.63
aug-cc-pVQZ				
NPA	−0.19	−0.66	−7.90	−10.00
VDD	−0.13	−0.24	−0.94	−1.14
6-31G**				
NPA	−0.18	−0.48	−7.04	−7.87
VDD	−0.11	−0.19	−0.80	−0.84
Mulliken	−0.02	−0.05	−0.31	−1.07

Table S6 RHF, SCGVB and CASSCF energies (in hartree) at the aug-cc-pVQZ C_{3v} geometries of M′AlH_3 (see Table S1). (Proportions of corresponding CASSCF(8,8) electron correlation energy are shown as percentages.)

Method	θ ~ +4°		θ ~ −35°	
	$MgAlH_3$	$CaAlH_3$	$MgAlH_3$	$CaAlH_3$
RHF	−443.2584671	−920.3973239	−443.2087862	−920.3778982
SCGVB[a]	−443.3156902 (98.9%)	−920.4584124 (98.9%)	−443.2911489 (98.7%)	−920.4572730 (98.9%)
SCGVB[b]	−443.3156652	−920.4583892	−443.2911410	−920.4572505
CASSCF(8,8)	−443.3163154	−920.4591111	−443.2922291	−920.4581244
CASSCF(8,11)	−443.3407315	−920.4835907	−443.3231489	−920.4866732

[a] Inactive orbitals taken from CASSCF(8,8)

[b] Inactive orbitals taken from CASSCF(8,11)

Table S7 SCGVB orbital overlaps for $MgAlH_3$ at its model $\theta = -35°$ geometry.

	φ1	φ2	φ3	φ4	φ5	φ6	φ7	φ8
φ1	1							
φ2	0.83	1						
φ3	0.13	0.20	1					
φ4	0.20	0.33	0.83	1				
φ5	0.13	0.20	0.13	0.20	1			
φ6	0.20	0.33	0.20	0.33	0.83	1		
φ7	0.12	0.29	0.12	0.29	0.12	0.29	1	
φ8	0.10	0.13	0.10	0.13	0.10	0.13	−0.25	1

Table S8 SCGVB orbital overlaps for $CaAlH_3$ at its model $\theta = +4°$ geometry.

	φ1	φ2	φ3	φ4	φ5	φ6	φ7	φ8
φ1	1							
φ2	0.81	1						
φ3	0.07	0.14	1					
φ4	0.14	0.30	0.81	1				
φ5	0.07	0.14	0.07	0.14	1			
φ6	0.14	0.30	0.14	0.30	0.81	1		
φ7	0.14	0.28	0.14	0.28	0.14	0.28	1	
φ8	0.02	0.03	0.02	0.03	0.02	0.03	0.48	1

Figure S1 Symmetry-unique SCGVB orbitals for $MgAlH_3$ (top row) and $CaAlH_3$ (bottom row) at model geometries.

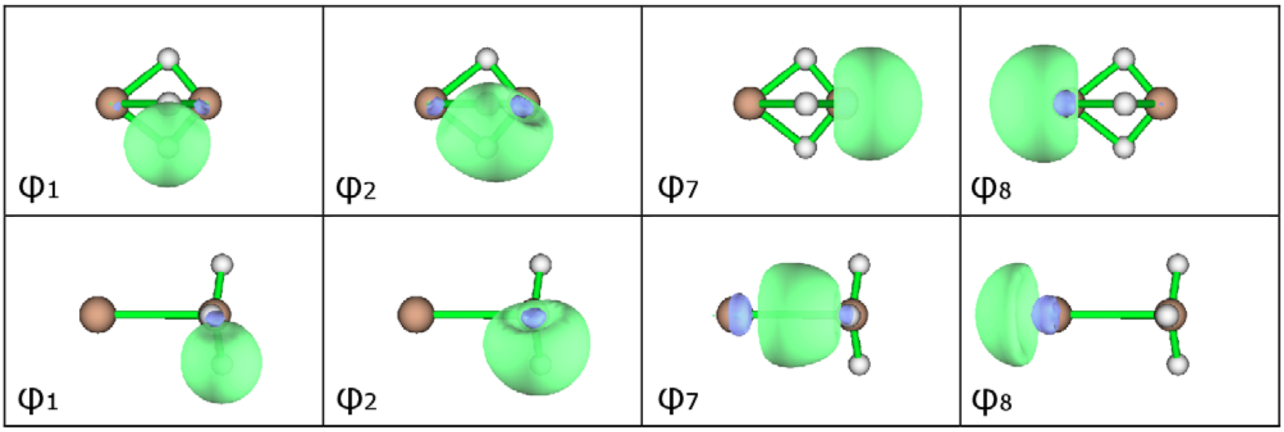

Table S9 Simple energy differences between the θ ~ −35° and θ ~ +4° CCSD(T)/aug-cc-pVQZ geometries, as calculated for a given molecule at various levels of theory using the aug-cc-pVQZ basis set. Negative values of Δ*E* indicate a preference for the θ ~ −35° geometry.

Method	Δ*E* (in kcal/mol)	
	$MgAlH_3$	$CaAlH_3$
RHF	31.2	12.2
SCGVB[a]	15.4	0.7
SCGVB[b]	15.4	0.7
CASSCF(8,8)	15.1	0.6
CASSCF(8,11)	11.0	−1.9
B3LYP	12.9	−6.8
CCSD	9.0	−16.6
CCSD(T)	4.6	−21.1

[a] Inactive orbitals taken from CASSCF(8,8)

[b] Inactive orbitals taken from CASSCF(8,11)
